# Encorafenib‐associated acantholytic dermatosis in collision with eccrine squamous syringometaplasia

**DOI:** 10.1111/ddg.70264

**Published:** 2026-06-04

**Authors:** Francisco Meraz‐Torres, Gabi Handgretinger, Andreas Meiwes, Derya Durak, Joana Schroth, Stephan Forchhammer

**Affiliations:** ^1^ Abteilung für Dermatologie Universitätsklinikum Tübingen, Deutschland

**Keywords:** acantholytic dermatosis, encorafenib, melanoma, squamous syringometaplasia

Dear Editors,

Some 47 % of cutaneous melanomas carry *BRAF* mutations that are involved in the mitogen‐activated protein kinase (MAPK) signaling pathway. One of the most common mutations here involves the exchange of valine for glutamic acid at codon 600.^1^ Encorafenib is a selective BRAF inhibitor and has recently been shown in phase III clinical trials to improve overall survival in patients with *BRAF*‐mutated stage IIIC and IV metastatic melanoma.^2^ However, targeted therapies such as application of BRAF inhibitors are associated with many dermatological side effects due to hyperactivation of intracellular signaling pathways.^3^ Adverse skin reactions occur in around 90 % of patients with melanoma treated with BRAF inhibitors. They mostly include acneiform exanthema in about 25‐85 % of cases, but also cutaneous squamous cell carcinoma and keratoacanthoma (18‐31 % and ≈ 45 %, respectively).^4^, ^5^

We report on a 66‐year‐old man with stage IV metastatic melanoma who was diagnosed with endonasal melanoma of the nasal cavity in December 2020. After radical excision and radiotherapy, adjuvant therapy with dabrafenib (Tafinlar®) and trametinib (Mekinist®) was carried out in July 2021 upon detection of a BRAF V600E mutation, but was discontinued in November 2021 due to an episode of documented polymyalgia rheumatica. Adjuvant therapy with nivolumab was initiated in July 2022 following lymphonodal tumor progression and subsequent radical neck dissection. As a significant worsening of the rheumatic symptoms and diffuse intrahepatic metastasis occurred after a short time under this treatment, the patient was switched to monotherapy with encorafenib (Braftovi®) 300 mg daily at the beginning of August 2022. Three weeks after switching therapy, the patient presented with itchy, exanthematous, erythematous and hyperkeratotic papules on the chest, back and armpit area, which quickly spread to the arms and legs (Figure 1). Two biopsies were performed with the initial suspected diagnosis of a lichenoid immunotherapy‐mediated drug reaction. The biopsy from the breast showed a focal acanthosis with a parakeratotic horny layer (Figure 2a). In the area of the acanthosis, the tissue showed a suprabasal cleft formation with numerous dyskeratotic keratinocytes without an interface reaction (Figure 2b). In the dermis, conspicuously widened eccrine sweat gland excretory ducts with a lining of enlarged keratinocytes with pale eosinophilic cytoplasm were seen. The keratinization was centrally parakeratotic. A moderately dense lymphohistiocytic inflammatory infiltrate was found in the periductal area (Figure 2c). Histologically, this shows a focal acantholytic dyskeratosis in collision with an eccrine squamous syringometaplasia (ESS). A second sample from the groin also showed ESS, whereas acantholytic changes were not seen.

**FIGURE 1:**
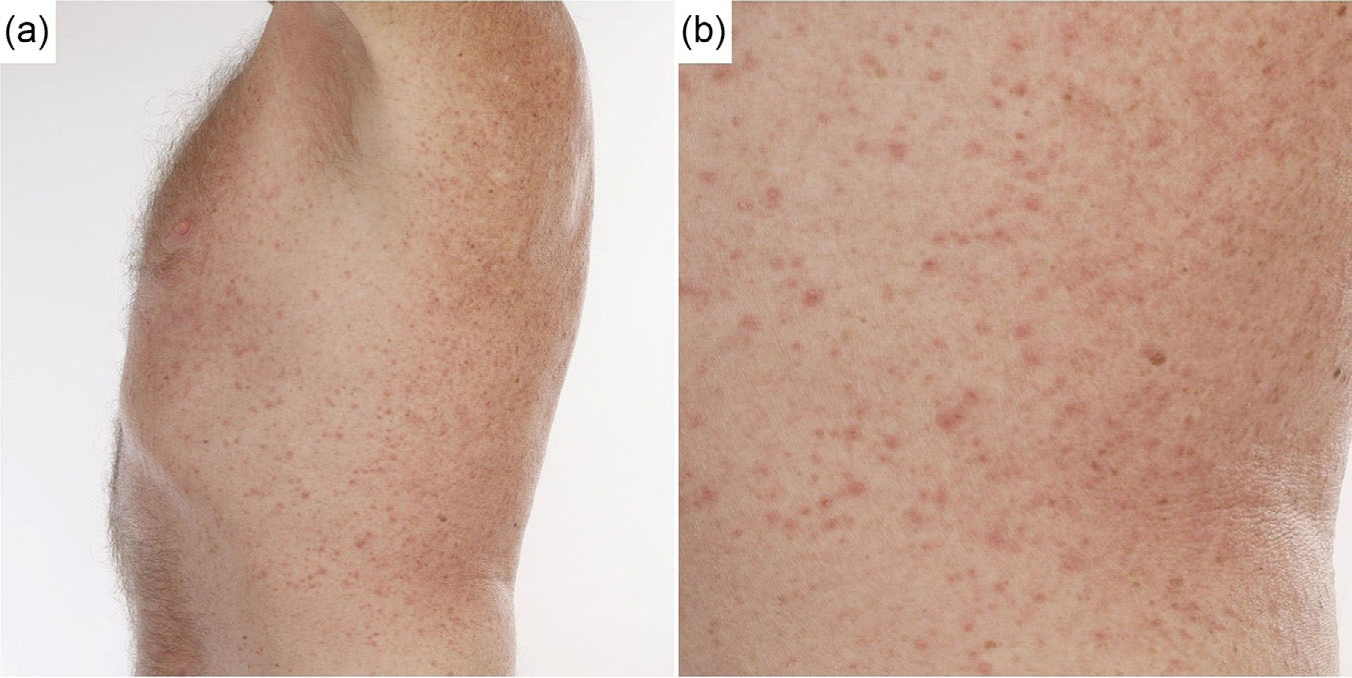
Photo documentation performed in August 2022. (a) Disseminated erythematous macules and hyperkeratotic papules, accentuated in the axillary region, back and trunk. (b) Magnification of a. Here, partly erythematous, partly light brownish papules with focal hyperkeratosis are evident.

**FIGURE 2:**
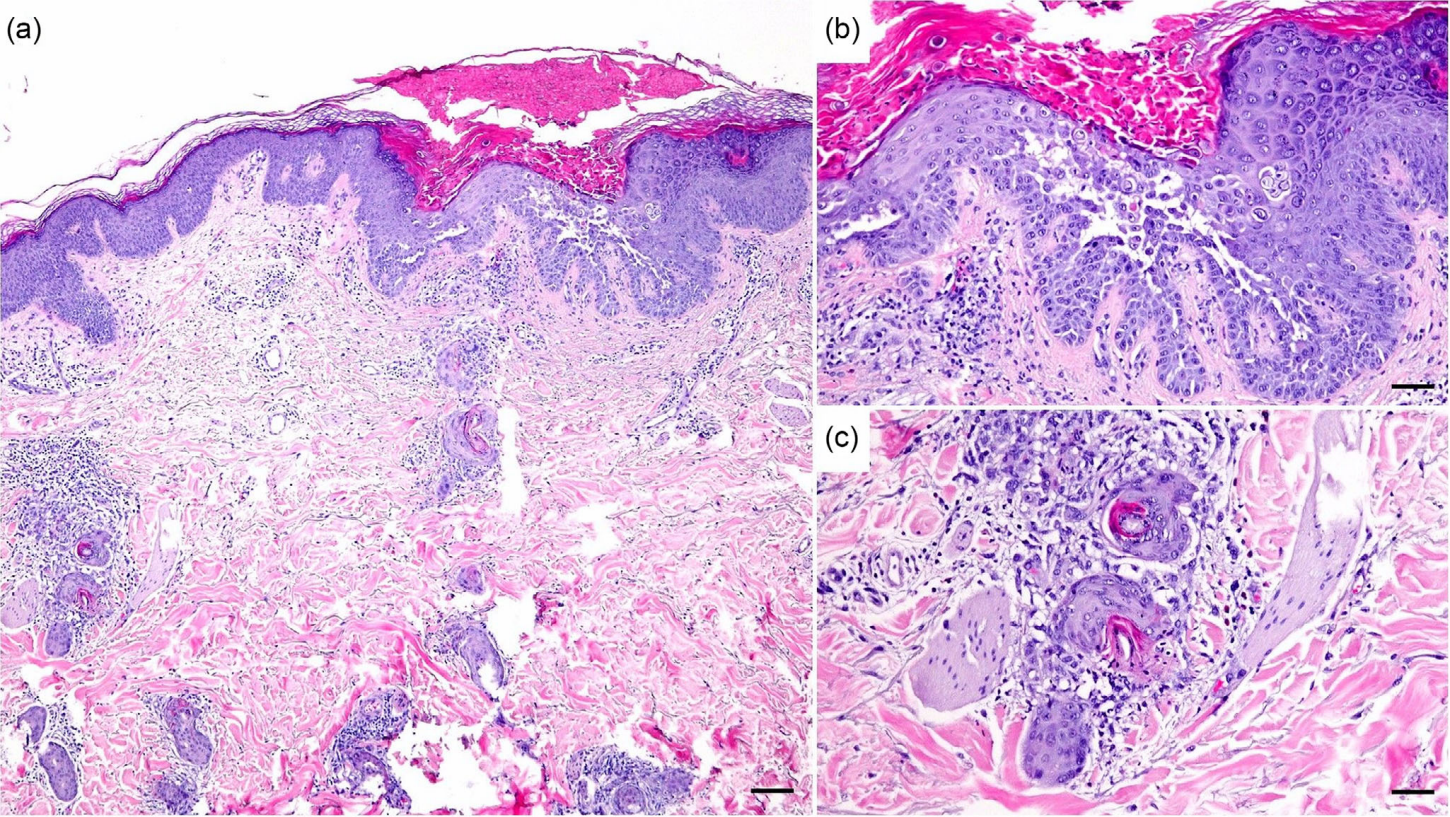
Punch biopsy from the chest, haematoxylin‐eosin staining. (a) Overview with focal acanthosis and acantholysis of the epidermis and underlying eccrine ducts with squamous syringometaplasia. Accompanying moderately dense lymphohistiocytic infiltrates. Scale = 150µm. (b) Magnification of the epidermal acanthotic zone with overlying parakeratosis. Pronounced suprabasal acantholysis zones with dyskeratotic keratinocytes are present. Scale = 60µm. (c) Eccrine squamous syringometaplasia with central keratinization. Scale = 60µm.

Due to the extensive skin findings, the cutaneous adverse event was classified as grade 3 according to the Common Terminology Criteria for Adverse Events (CTCAE version 5.1). Treatment with encorafenib was switched to a targeted combination therapy with the BRAF inhibitor dabrafenib and the MEK inhibitor trametinib. Healing was observed within a few weeks under this treatment, with the additional use of systemic retinoids (acitretin 10 mg 0‐1‐0), topical steroid cream and urea‐containing basic care.

In summary, this is a very rare focal acantholytic dermatosis in collision with eccrine squamous syringometaplasia under monotherapy with a BRAF inhibitor, which has not yet been described with encorafenib. In melanoma patients with a BRAF mutation, the MAPK signaling pathway of melanoma cells is constitutively activated. In this respect, the mutated BRAF protein acts as a protooncogene that induces the proliferation of melanoma cells.^6^ Therefore, BRAF inhibition leads to a significant shrinkage of the tumor. However, BRAF inhibitor monotherapy can lead to paradoxical MAPK activation in cells of the epidermis as well as the eccrine epithelium. This paradoxical activation is the cause of cutaneous side effects of BRAF inhibitors and has been shown for cutaneous squamous cell carcinomas and keratoacanthomas.^7^ These paradoxical activations do not occur under combination therapy with MEK inhibitors, which is why corresponding cutaneous side effects are rarely observed. In ESS, the cubic epithelial cells typical of eccrine sweat gland ducts are transformed into squamous epithelial cells. This adaptive cellular process occurs in response to various stimuli.^8^ ESS presents clinically as an erythematous papular exanthema that usually occurs a few days after the initiation of some toxic agents or radiation therapy.^9^ Focal acantholytic dyskeratosis is a characteristic histologic pattern that can occur in both genodermatoses such as Darier's disease and inflammatory dermatoses such as Grover's disease. However, it has also been described under monotherapy with vemurafenib. A link to a paradoxical MAPK activation is also suspected here.^10^ In addition, cases have been described in which focal acantholytic dyskeratosis was incidentally found in various skin lesions.^11^ Due to the rapid healing after switching to BRAF/MEK combination therapy, we assume that both ESS and acantholytic dyskeratosis occurred during monotherapy with encorafenib as a result of paradoxical MAPK activation.

## CONFLICT OF INTEREST STATEMENT

None.
